# Tumor exosome promotes Th17 cell differentiation by transmitting the lncRNA CRNDE-h in colorectal cancer

**DOI:** 10.1038/s41419-020-03376-y

**Published:** 2021-01-25

**Authors:** Junfeng Sun, Haowei Jia, Xingqi Bao, Yue Wu, Tianyu Zhu, Ruixin Li, Hongchao Zhao

**Affiliations:** Department of Gastrointestinal Surgery, The First Affiliated Hospital of Zhengzhou University, Zhengzhou, 450052 Henan China

**Keywords:** Cancer, Cell biology

## Abstract

The T helper 17 (Th17) cells in tumor microenvironment play an important role in colorectal cancer (CRC) progression. This study investigated the mechanism of Th17 cell differentiation in CRC with a focus on the role of tumor exosome-transmitted long noncoding RNA (lncRNA). Exosomes were isolated from the CRC cells and serum of CRC patients. The role and mechanism of the lncRNA CRNDE-h transmitted by CRC exosomes in Th17 cell differentiation were assessed by using various molecular biological methods. The serum exosomal CRNDE-h level was positively correlated with the proportion of Th17 cells in the tumor-infiltrating T cells in CRC patients. CRC exosomes contained abundant CRNDE-h and transmitted them to CD4^+^ T cells to increase the Th17 cell proportion, RORγt expression, and IL-17 promoter activity. The underlying mechanism is that, CRNDE-h bound to the PPXY motif of RORγt and impeded the ubiquitination and degradation of RORγt by inhibiting its binding with the E3 ubiquitin ligase Itch. The in vivo experiments confirmed that the targeted silence of CRNDE-h in CD4^+^ T cells attenuated the CRC tumor growth in mice. The present findings demonstrated that the tumor exosome transmitted CRNDE-h promoted Th17 cell differentiation by inhibiting the Itch-mediated ubiquitination and degradation of RORγt in CRC, expanding our understanding of Th17 cell differentiation in CRC.

## Introduction

Colorectal cancer (CRC) is the most common cancer in the digestive system and is the second leading cause of cancer-related death; with a mortality rate of 8%-9%^1,2^. CRC development and progression are correlated with diverse factors, and many studies have shown that tumor immune microenvironment is closely correlated with the development and progression of CRC^3,4^. The increased proportion of T helper 17 (Th17), a subset of T cells derived from CD4^+^ T cells, in a tumor immune microenvironment have been reported to be associated with the occurrence and development of malignant tumors^5–7^. Besides, studies have shown that a large number of differentiated and mature Th17 cells are accumulated in colorectal tissues of patients with CRC, which can produce interleukin 17 (IL-17) to promote the development and progression of CRC via multiple mechanisms^8,9^. On the basis of the role of Th17 cells in CRC, blocking the differentiation of Th17 cells from CD4^+^ T cells may be a new therapeutic method against CRC. However, the mechanism of Th17 cell differentiation in CRC remains largely unknown.

Exosomes, a subset of small extracellular vesicles, contain many bioactive molecules, such as proteins, lipids, microRNAs, and long noncoding RNAs (lncRNAs), and these exosomes function as intercellular shuttles that to facilitate interactions with neighboring cells by transmitting those bioactive molecules^10–13^. Various types of cells, such as mesenchymal stem cells, dendritic cells, and tumor cells, can secrete exosomes^14^. It has been found that tumor-derived exosomes can alter a tumor microenvironment via their involvement in angiogenesis and in the regulation of matrix cells, and remodeling of extracellular matrix^15^. Moreover, studies have reported that tumor exosomes can facilitate Th17 cell differentiation in many cancers, including gastric cancer and CRC^6,16^. However, the mechanism by which tumor-exosome regulates Th17 cell differentiation in CRC has not yet been fully determined.

LncRNAs, a class of transcripts longer than 200 nucleotides with a limited or without protein-coding function, are involved in many diseases owing their modulating effect toward diverse biological processes, such as cell proliferation, apoptosis, and differentiation^17^. More importantly, many lncRNAs, such as lncRNA MEG3, lncRNA NEAT1, and lncRNA H19, have been proven to be tightly linked to Th7 cell differentiation^18–20^. Colorectal neoplasia differentially expressed (CRNDE), an lncRNA located on chromosome 16, is highly expressed in multiple cancers, and it plays indispensable roles in the development of different types of cancer, including CRC^21^. Many isoforms of CRNDE transcripts are significantly upregulated in CRC cells and tissues^22^. Among these upregulated CRNDE isoforms, CRNDE-h is found to be stable in CRC cell-derived serum exosomes and is a potential marker for the early diagnosis and prognosis of CRC^23^. The literature has shown that the CRNDE-h transmitted by CRC cell-derived exosomes is significantly correlated with the growth, metastasis, and poor prognosis^24,25^. However, whether CRNDE-h is involved in the regulation of CRC exosomes in Th17 cell differentiation remains unknown.

In this work, we investigated the mechanism of Th17 cell differentiation in CRC with a focus on the tumor exosome-transmitted CRNDE-h. We first detected the abundance of CRNDE-h in the serum exosomes of CRC patients and CRC cell-secreted exosomes. Then, we confirmed whether the CRNDE-h transmitted by the CRC exosomes to CD4^+^ T contributing to the differentiation of CD4^+^ T cells into Th17 cells. Last, we demonstrated the mechanism that CRNDE-h bound to the PPXY motif of the RAR-related orphan receptor γt (RORγt) and impeded its ubiquitination and degradation by inhibiting the binding of RORγt to the E3 ubiquitin ligase Itch. Taken together, this study determined an underlying mechanism of Th17 cell differentiation in CRC.

## Results

### Serum exosomal CRNDE-h level was positively correlated with Th17 cell proportion in tumor-infiltrating T cells

**Fig. 1:**
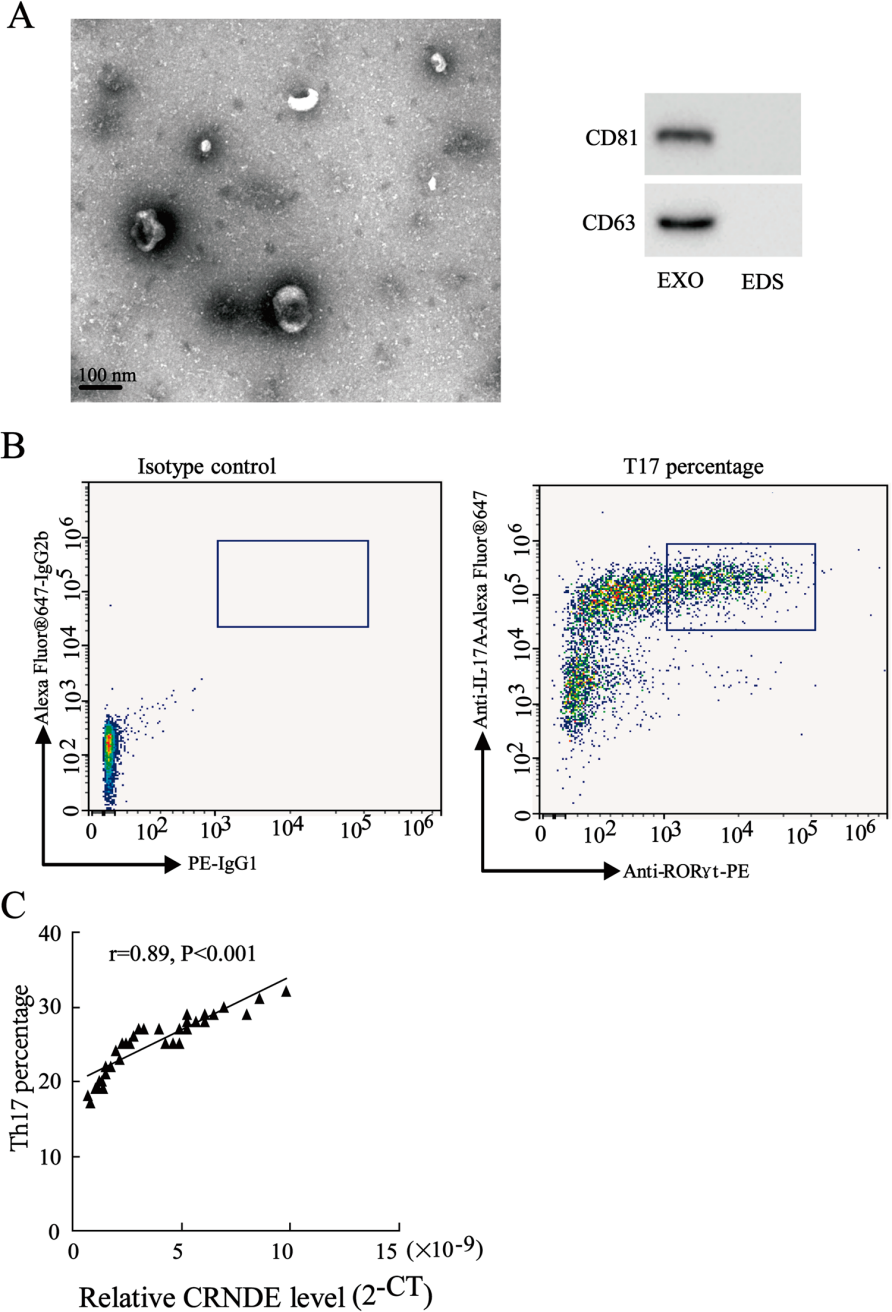
The correlation between the CRNED-h expression in serum-exosome and Th17 cell proportion. **A** The characterization of serum-derived exosome was determined by transmission electron micrograph, and western blot analysis was used to detect positive exosomal markers. **B** The proportion of Th17 cells in the tumor-infiltrating T cells isolated from CRC patients was detected by flow cytometry. **C** The correlation between the expression of CRNDE-h in the serum-derived exosome from 42 CRC patients and the proportion of Th17 cells in tumor-infiltrating T cells. EXO exosome, EDS exosome depleted supernatant.

To determine the clinical relevance between serum exosomal CRNDE-h level and Th17 cell proportion, we isolated serum exosome and tumor infiltrating T cells from 42 CRC patients. The electron microscopic analysis revealed that the size of the serum exosomes ranged from 30 nm to 150 nm. Moreover, the Western blot analysis revealed that the serum exosomes positively expressed the exosomal-specific marker proteins CD81 and CD63 (Fig. 1A). Through a flow cytometric analysis, we found that there were a high proportion of Th17 cells in the tumor-infiltrating T cells of our CRC patients (Fig. 1B). Moreover, the correlation analysis showed a positive correlation between the Th17 cells in the tumor-infiltrating T cells and the exosomal CRNDE-h expression in the CRC patients (Fig. 1C), implying a clinical relevance between exosomal CRNDE-h level and Th17 cell proportion in CRC patients.

### CRC cell-derived exosomes transmitted CRNDE-h to naive CD4^+^ T cells

**Fig. 2:**
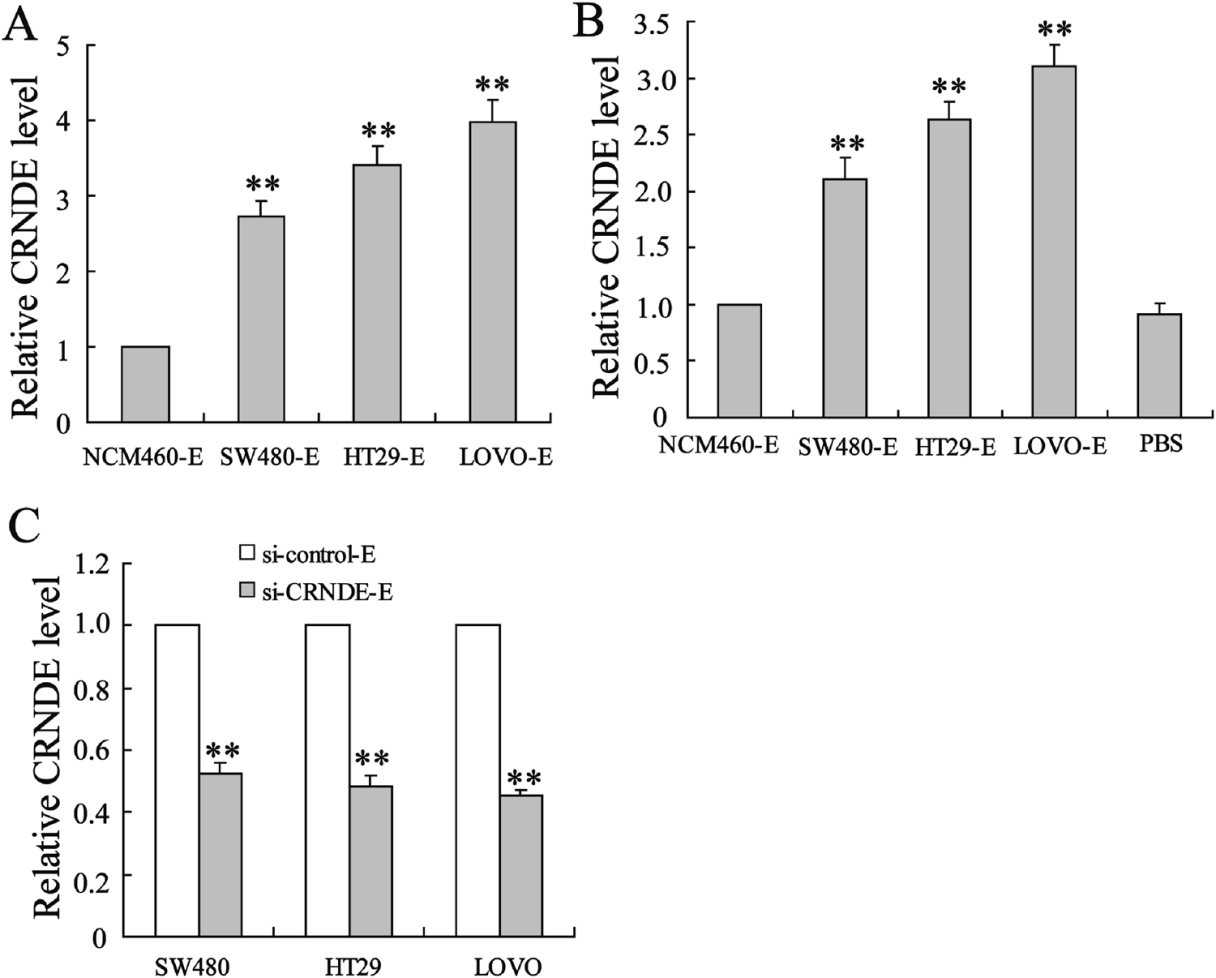
The effects of CRC cell exosome on the expression of CRNDE-h in CD4^+^ T cells. **A** The expressions of CRNDE-h in SW480-exosome (SW480-E), HT29-exosome (HT29-E), LOVO-exosome (LOVO-E), (LOVO-E), and normal colon epithelial cell-exosome (NCM460-E) were detected by qRT-PCR. **B** The SW480-E, HT29-E, LOVO-E, and NCM460-E (20 μg/mL) were co-cultured with naive CD4^+^ T cells isolated from the PMBCs of healthy subjects for 24 h. Then, the expression of CRNDE-h in T cells was detected by qRT-PCR. The “μg/mL” in this study means the protein concentration of exosomes. **C** CRC cells (SW480, HT29, and LOVO) were transfected with si-CRNDE-h (or si-control), and then used to isolate exosomes. Exosomes were co-cultured with naive CD4^+^ T cells isolated from the PMBCs of healthy subjects. Then, the expression of CRNDE-h in T cells was detected by qRT-PCR. E: exosome. **p < 0.01 vs. NCM460-E or si-control-E.

To investigate whether CRC cell exosomes can transmit CRNDE-h to naive CD4^+^ T cells, we first detected the expressions of CRNDE-h in CRC cell exosomes. As shown in Fig. 2A, the expressions of CRNDE-h in the exosomes secreted by the CRC cell lines, namely SW480, HT-29, and LOVO, were significantly higher than that in the exosome secreted by the normal colon epithelial cell line NCM460. Second, we co-cultured the CRC cell exosomes with naive CD4^+^ T cells for 24 h, and then detected the expression of CRNDE-h in the CD4^+^ T cells. We compared CRNDE-h levels in CD4^+^ T cells not co-cultured with exosomes (treated with PBS) and CD4^+^ T cells co-cultured with NCM460-E. The results showed that CRNDE-h level in the PBS group was slightly reduced when compared with the NCM460-E group, but there was no statistical significance, as shown in Fig. 2B. Expectedly, after co-culture with the SW480, HT-29, and LOVO-derived exosomes, the levels of CRNDE-h in the CD4^+^ T cells were significantly elevated (Fig. 2B). Third, the levels of CRNDE-h in CRC cells were depleted by si-CRNDE-h transfection, and then the exosomes were isolated from the CRNDE-h-depleted CRC cells to be co-cultured with naive CD4^+^ T cells. Our results showed that CRNDE-h level in the CD4^+^ T cells co-cultured with the CRNDE-h-depleted CRC cell-exosome was evidently lower than that in the CD4^+^ T cells co-cultured with normal CRC cell exosomes (Fig. 2C). These data confirmed that CRC cell exosomes could transmit CRNDE-h to CD4^+^ T cells.

### CRC cell exosomes contributed to Th17 cell differentiation by transmitting CRNDE-h

**Fig. 3:**
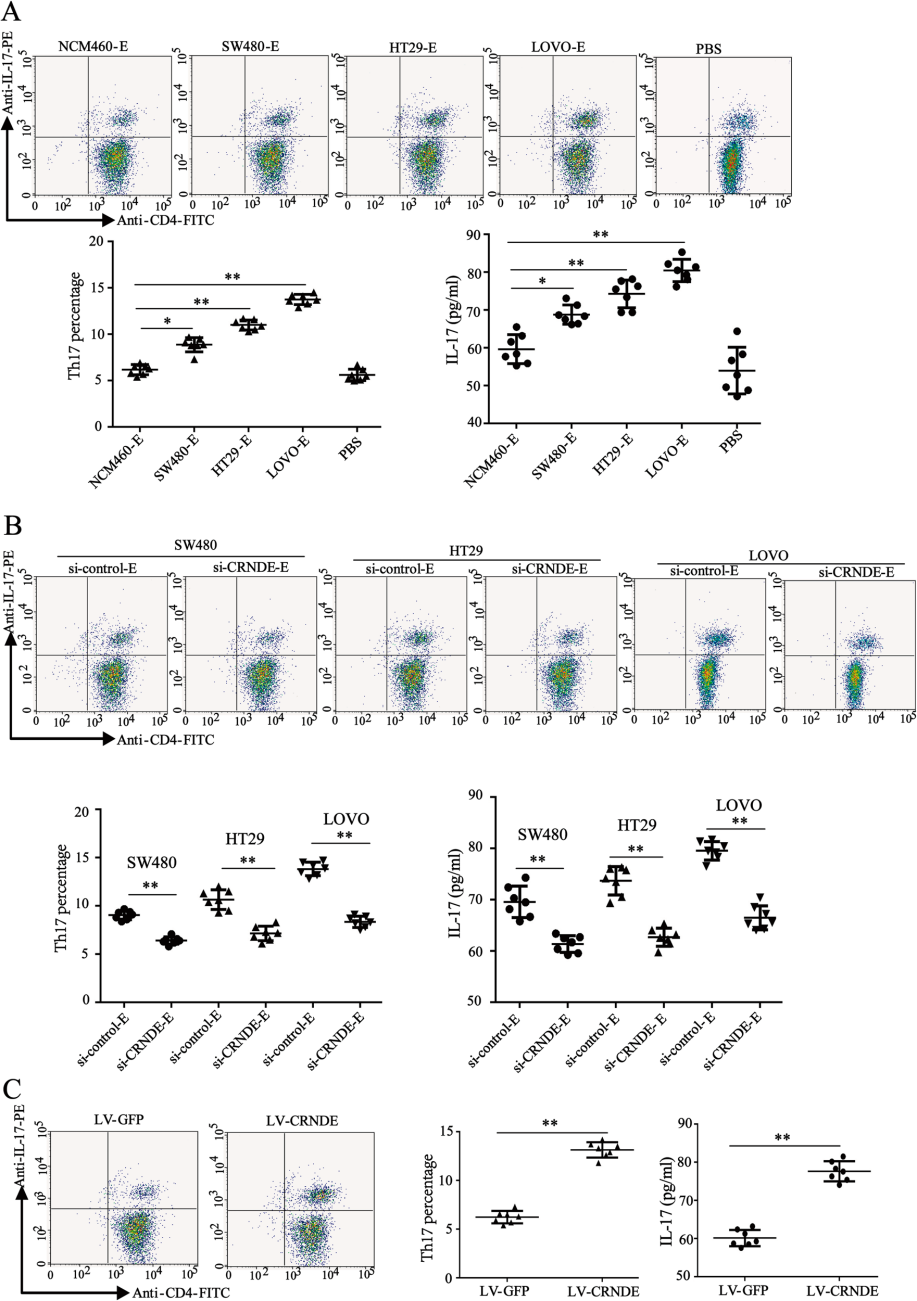
CRNDE-h mediated the role of CRC cell-exosome in promoting Th17 cell differentiation. **A** PBMCs isolated from healthy subjects were cultured under the condition of Th17 cell differentiation induction and treated with CRC cell-exosome (SW480-E, HT29-E, and LOVO-E). Then, the proportion of Th17 cells was detected by flow cytometry, and the content of IL-17 in the cell culture supernatant was detected by ELISA assay. **B** Healthy PBMCs were cultured under the condition of Th17 cells differentiation induction and treated with exosome derived by CRC cells (SW480, HT29, and LOVO) transfected with si-CRNDE-h (or si-control). Then, the proportion of Th17 cells was detected by flow cytometry, and the content of IL-17 in the cell culture supernatant was detected by ELISA assay. **C** Healthy PBMCs were transfected with lentiviral (LV) vectors carrying CRNDE-h (LV-CRNDE-h) or green fluorescent protein (LV-GFP), and then cultured under the condition of Th17 cell differentiation induction. The proportion of Th17 cells was detected by flow cytometry and the content of IL-17 in the cell culture supernatant was detected by ELISA assay. E: exosome. *p < 0.05, **p < 0.01 vs. NCM460-E, si-control-E, or LV-GFP.

To determine the role of CRC cell exosomes in Th17 cell differentiation in vitro, we co-cultured human healthy PBMCs with CRC cell exosomes under the condition of Th17 cell differentiation. The conditions of Th17 cell differentiation: Healthy PBMCs were cultured under the inductive condition of Th17 cells (25 μL/well Human T-Activator CD3/CD28 Dynabeads in 24-well plates, IL-6 100 ng/mL, and TGF-β 20 ng/mL) and treated with individual exosomes or PBS (the absence of exosome) for 5 d. As shown in Fig. 3A, the co-culture with SW480, HT-29, or LOVO-derived exosomes increased both IL-17 content in cell culture supernatant and the Th17 cell proportion. To determine whether CRC cell exosomes regulated Th17 cell differentiation by transmitting CRNDE-h, we co-cultured human healthy PBMCs with CRNDE-h-depleted CRC cell exosomes under the condition of Th17 cell differentiation. The results of the flow cytometry and ELISA assays showed that the co-culture with the CRNDE-h-depleted CRC cell exosomes reduced both IL-17 content in the cell culture supernatant and the Th17 cell proportion (Fig. 3B). To further verify the role of CRNDE-h in Th17 cell differentiation, we induced healthy human PBMCs overexpressed CRNDE-h through LV-CRNDE-h transfection, and then we cultured them under the condition of Th17 cell differentiation. Our results illustrated that overexpression of CRNDE-h augmented both IL-17 content in the cell culture supernatant and the Th17 cell proportion (Fig. 3C). These data collectively suggested that CRC cell exosomes facilitated Th17 cell differentiation by transmitting CRNDE-h.

### CRC cell-exosomal CRNDE-h increased the RORγt expression and IL-17 promoter activity

**Fig. 4:**
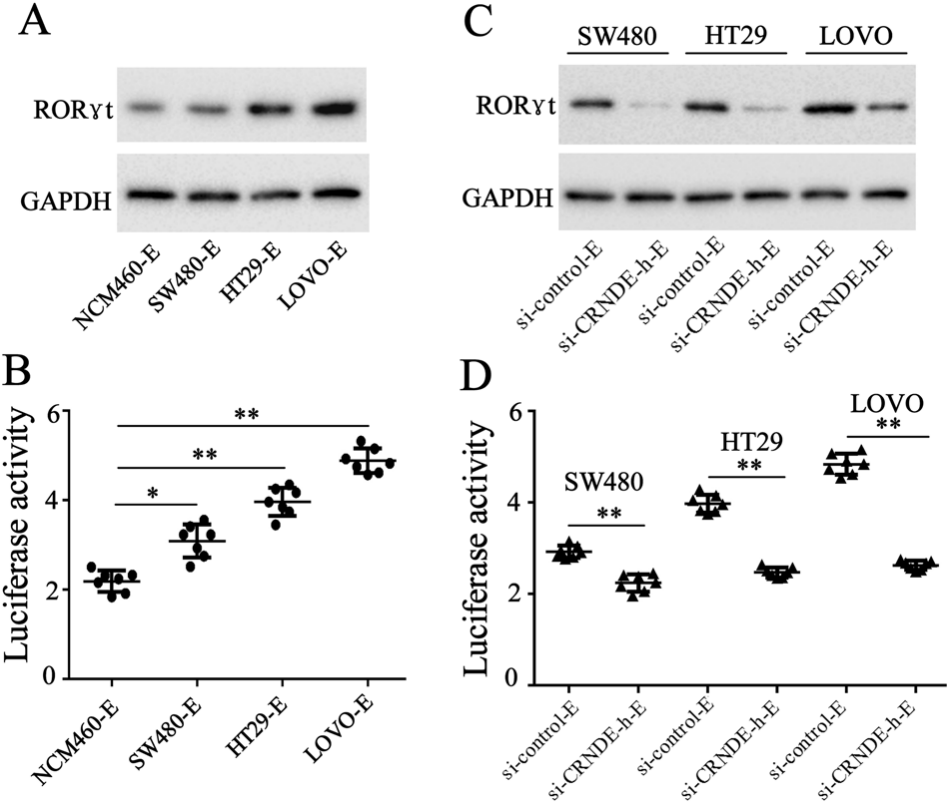
CRC cell-derived exosomal CRNDE-h increased the expression and activity of RORγt. **A** Naive CD4^+^ T cells treated with CRC cell-exosome (SW480-E, HT29-E, and LOVO-E) for 72 h, and then the protein level of RORγt was detected by western blot. **B** Naive CD4^+^ T cells treated with exosome derived by CRC cells (SW480, HT29, and LOVO) were transfected with si-CRNDE-h (or si-control) for 72 h. Later, the protein level of RORγt was detected by western blot. **C** Jurkat cells were transfected with IL-17 promoter-driven luciferase vector, and then treated with CRC cell-exosome (SW480-E, HT29-E, and LOVO-E) for 72 h. Later, luciferase activity was detected. **D** Jurkat cells were transfected with IL-17 promoter-driven luciferase vector, and then treated with exosome derived by CRC cells (SW480, HT29, and LOVO) transfected with si-CRNDE-h (or si-control) for 72 h. Later, luciferase activity was detected. E: exosome. *p < 0.05, **p < 0.01 vs. NCM460-E or si-control-E.

It has been determined that the transcription factor RORγt can induce the differentiation of naive CD4^+^ T cells into Th17 cells by binding to the IL-17 promoter to promote IL-17 secretion^26,27^. Considering the important role of RORγt in Th17 cell differentiation, we detected the effects of CRC cell exosomes on RORγt expression and IL-17 promoter activity. From the western blot analysis results, we found that SW480, HT-29, or LOVO-derived exosomes increased the protein level of RORγt in CD4^+^ T cells (Fig. 4A). Also, the luciferase reporter gene assay revealed that the CRC cell exosomes increased the promoter activity of IL-17 in Jurkat cells (Fig. 4B). To determine whether the effects of CRC cell exosomes on RORγt expression and promoter activity were correlated with CRNDE-h, we detected the effects of CRNDE-h-depleted CRC cell exosomes on RORγt expression and IL-17 promoter activity. As shown in Fig. 4C, D, compared with the normal CRC cell exosomes, the CRNDE-h-depleted CRC cell exosomes reduced the protein level of RORγt in CD4^+^ T cells and the promoter activity of IL-17 in Jurkat cells. These results indicated that CRC cell exosomal CRNDE-h increased RORγt expression and IL-17 promoter activity in naive CD4^+^ T cells.

### CRNDE-h bound to RORγt at the PPXY motif

**Fig. 5:**
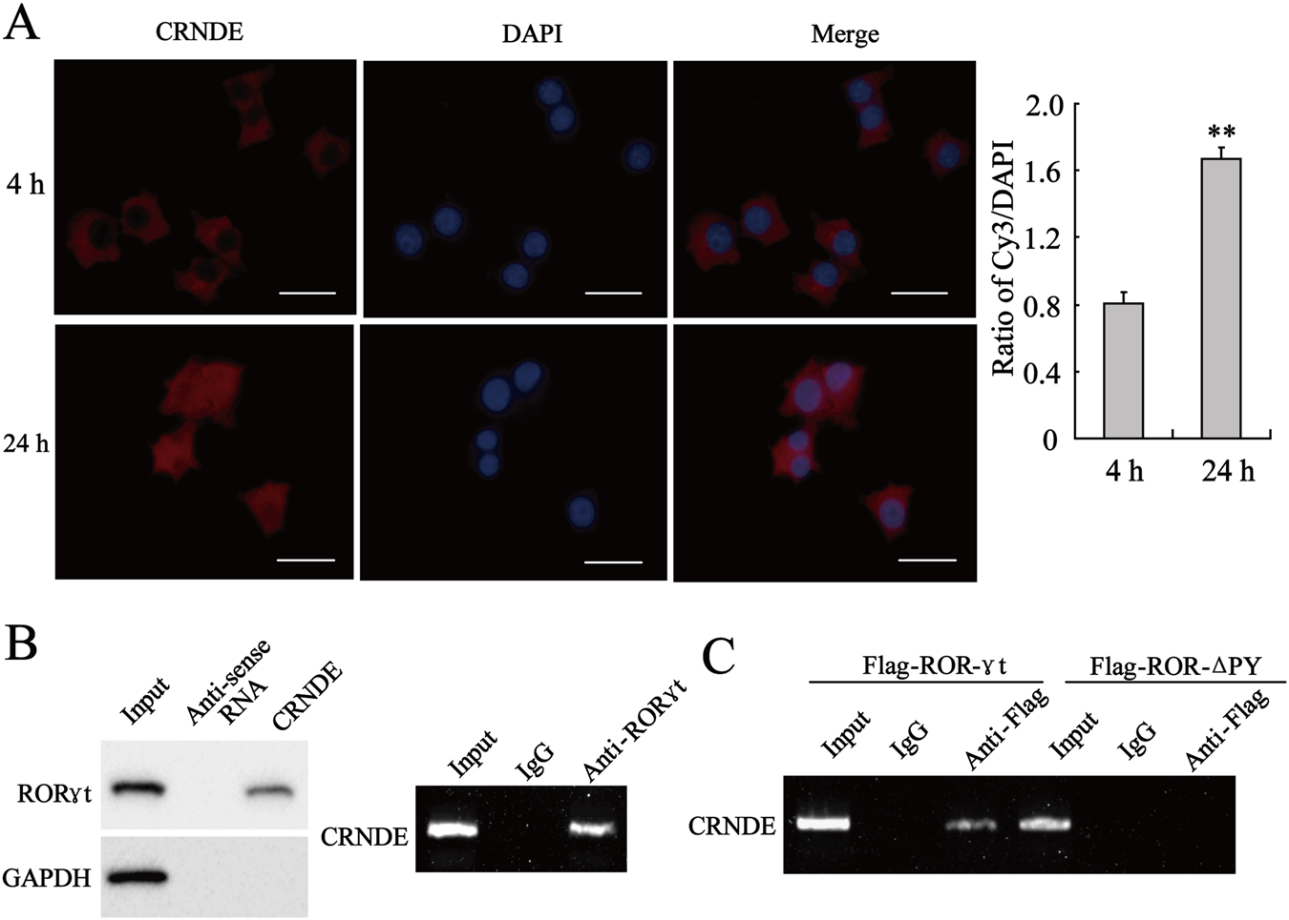
CRNDE-h bond to RORγt. **A** Naive CD4^+^ T cells were treated with CRC cell-exosome (LOVO-E) for indicated times (4 h and 24 h). The distribution of CRNDE-h in cells was displayed by FISH. Bars = 5 μm. The ratio of Cy3/DAPI was analyzed. **p < 0.01 vs. 4 h. **B** Naive CD4^+^ T cells were treated with CRC cell-exosome (LOVO-E) for 72 h. RNA pull-down (left): the enrichment of RORγt in the products pulled-down by CRNDE-h or its antisense RNA. RIP (right): the enrichment of CRNDE in the products immunoprecipitated by anti-RORγt or anti-IgG. **C** The 293 A cells were co-transfected with CRNDE-h overexpression vector (LV-CRNDE-h) and Flag-tagged wild type RORγt (Flag-RORγt) or a deletion mutant of RORγt that lacked amino acids 476–479 of the PPXY motif (Flag-ROR-ΔPY). RIP: the enrichment of CRNDE-h in the products immunoprecipitated by anti-RORγt or anti-IgG.

To explore the mechanism by which CRNDE-h regulated RORγt expression in CD4^+^ T cells, we first investigated the cellular location of CRNDE-h in LOVO-derived exosomes co-cultured with naive CD4^+^ T cells. As shown in Fig. 5A, CRNDE-h was mainly located in the cytoplasm of CD4^+^ T cells 4 h after co-culture and in the cytoplasm and nucleus of CD4^+^ T cells 24 h after co-culture. Subsequently, we verified whether CRNDE-h could bind to RORγt. The RNA pull-down results revealed that RORγt protein was enriched in the products pulled down by CRNDE-h, and the RIP assay showed that CRNDE-h was enriched in the products immunoprecipitated by the RORγt antibody (Fig. 5B), indicating that CRNDE-h could bind to RORγt. Given that there is a highly conserved PPXY motif in the amino acid sequence of RORγt in different species^27^, 293 A cells were co-transfected with LV-CRNDE-h and Flag-tagged RORγt (Flag-RORγt) or with Flag-tagged mutant RORγt that lacked the PPXY motif at the 476–479 amino acids residues (Flag-RORγt-ΔPY) to investigate whether CRNDE-h bound to RORγt at its PPXY motif. Interestingly, RIP assay showed that CRNDE-h was enriched in the products immunoprecipitated by Flag antibody in the Flag-RORγt-transfected cells, whereas no enrichment of CRNDE-h was observed in the products immunoprecipitated by Flag antibody in the Flag-RORγt-ΔPY-transfected cells (Fig. 5C). Together, these data revealed that CRNDE-h bound to RORγt at its PPXY motif.

### CRNDE-h hindered RORγt ubiquitination by inhibiting the binding of RORγt to Itch

**Fig. 6:**
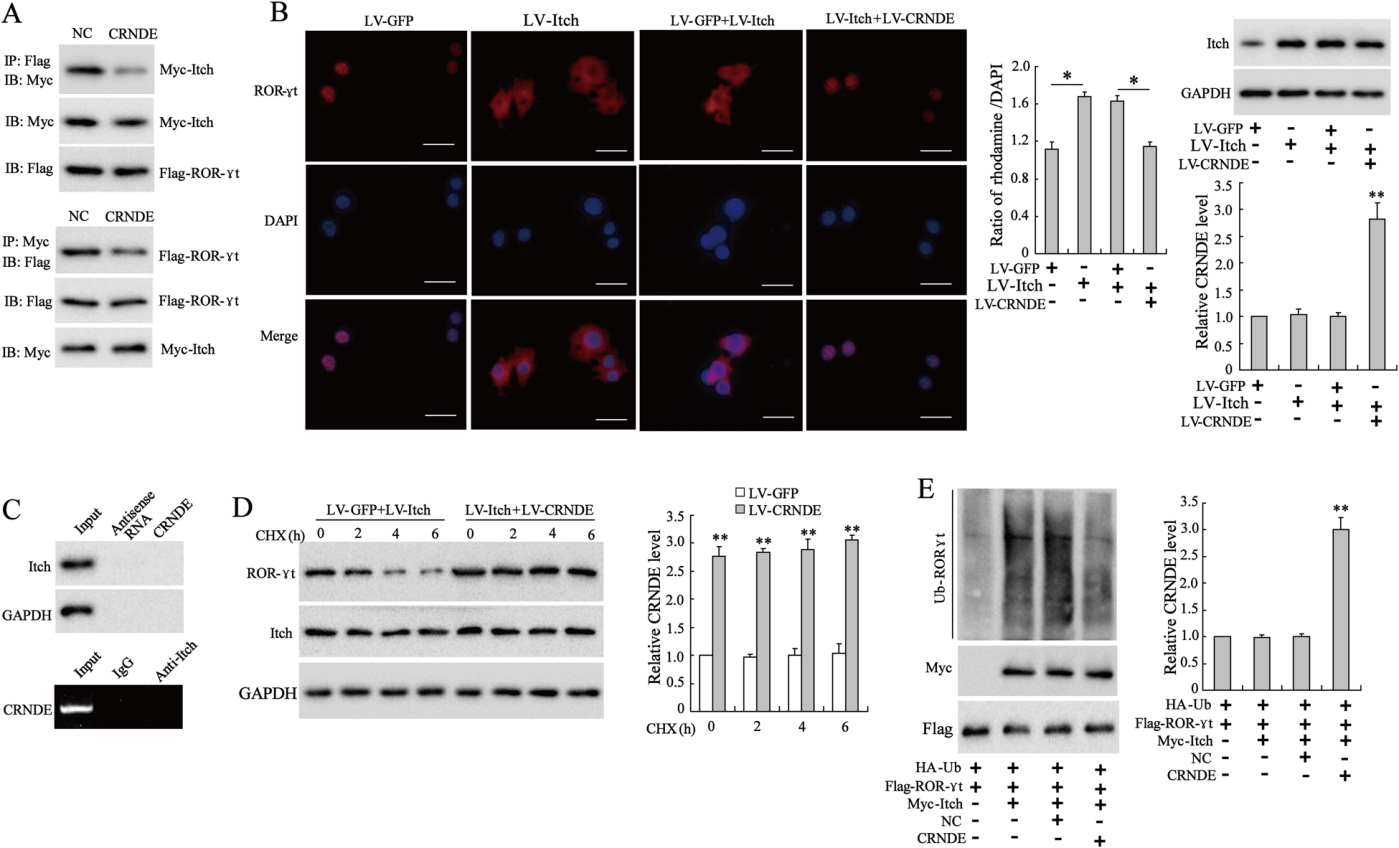
CRNDE-h inhibited Itch-mediated RORγt ubiquitination and degradation by blocking the binding of Itch to RORγt. **A** The 293 A cells were co-transfected CRNDE-h overexpression vector (LV-CRNDE-h), Flag-tagged RORγt (Flag-RORγt), and Myc-tagged Itch (Myc-Itch). Co-IP: the enrichment of Myc-Itch in the products immunoprecipitated by Flag antibody (left); the enrichment of Flag-ROR-γ in the products immunoprecipitated by Myc antibody (right). IP: immunoprecipitation; IB: immunoblotting; NC: negative control of LV-CRNDE-h. **B** Tumor-infiltrating T cells were transfected with LV-Itch alone or co-transfected with LV-Itch and LV-CRNDE-h. The location of RORγt in cells was displayed by immunofluorescent staining. Bars = 5 μm. The ratio of rhodamine/DAPI was analyzed. *p < 0.05 vs. LV-GFP or LV-Itch+LV-CRNDE-h. Western blot was used to detect the expression of Itch, and qRT-PCR was used to detect the level of CRNDE-h. **p < 0.01 vs. LV-Itch+LV-CRNDE-h. **C** The RIP and RNA pull-down assays were performed in tumor-infiltrating T cells. RNA pull-down (upper): the enrichment of Itch in the products pulled-down by CRNDE-h or its antisense RNA. RIP (lower): the enrichment of CRNDE-h in the products immunoprecipitated by anti-Itch or anti-IgG. **D** Tumor-infiltrating T cells were co-transfected with LV-Itch and LV-CRNDE-h (or LV-GFP), and then were treated with cycloheximide (CHX) for indicated times (0, 2, 4, and 6 h). Later, the protein levels of RORγt and Itch were detected by western blot, and the level of CRNDE-h was detected by qRT-PCR. **p < 0.01 vs. LV-GFP. **E** The 293 A cells were co-transfected with Myc-tagged Itch (Myc-Itch), Flag-tagged RORγt (Flag-RORγt), Lv-CRNDE-h, and HA-tagged ubiquitin (HA-Ub). Then the ubiquitination of RORγt was detected by immunoprecipitation assay. The level of CRNDE-h was detected by qRT-PCR. **p < 0.01 vs. NC.

Considering that the E3 ubiquitin ligase Itch can target PPXY motif of RORγt to mediate the ubiquitination of RORγt^27^, we wondered whether CRNDE-h could bind to Itch and affect the Itch-mediated ubiquitination of RORγt. To find an answer, we first co-transfected Flag-RORγt, Myc-tagged Itch (Myc-Itch), and LV-CRNDE-h into 293 A cells, and then we performed a Co-IP assay to assess the effect of CRNDE-h overexpression on the binding of Itch to RORγt. The results revealed that the overexpression of CRNDE-h inhibited the binding capacity of Itch to RORγt (Fig. 6A). Then, we investigated the effects of Itch and CRNDE-h on the nuclear translocation of RORγt in tumor-infiltrating T cells. After transfected with LV-Itch, the expression of Itch in tumor-infiltrating T cells was improved when compared with LV-GFP group. The level of CRNDE-h was also upregulated after transfected with LV-CRNDE-h (Fig. 6B). From the immunofluorescence staining results, we found that the LV-Itch-induced Itch overexpression contributed to the nuclear export of RORγt, whereas CRNDE-h overexpression induced by LV-CRNDE-h abolished this effect (Fig. 6B). Next, we verified whether CRNDE-h could also bind to Itch in tumor-infiltrating T cells by using RIP and RNA pull-down assays. As shown in Fig. 6C, there was no enrichment of Itch in the products pulled down by CRNDE-h, nor was there any enrichment of CRNDE-h in the products immunoprecipitated by Itch antibody, confirming that CRNDE-h could not bind to Itch. Furthermore, we studied whether CRNDE-h could affect the Itch-mediated degradation of RORγt in the tumor-infiltrating T cells. The western blot analysis revealed that the overexpression of CRNDE-h impeded the Itch-mediated degradation of RORγt protein in the presence of the protein synthesis inhibitor cycloheximide (CHX) (Fig. 6D). Finally, we evaluated the effect of CRNDE-h on the Itch-mediated ubiquitination of RORγt in 293 A cells. The ubiquitination experiments showed that Itch mediated the ubiquitination of RORγt, whereas CRNDE-h overexpression hindered the Itch-mediated ubiquitination of RORγt (Fig. 6E). These results demonstrated that CRNDE-h blocked RORγt ubiquitination and degradation by suppressing the binding of RORγt to Itch.

### The silence of CRNDE-h in CD4^+^ T cells inhibited the CRC tumor growth in mice

**Fig. 7:**
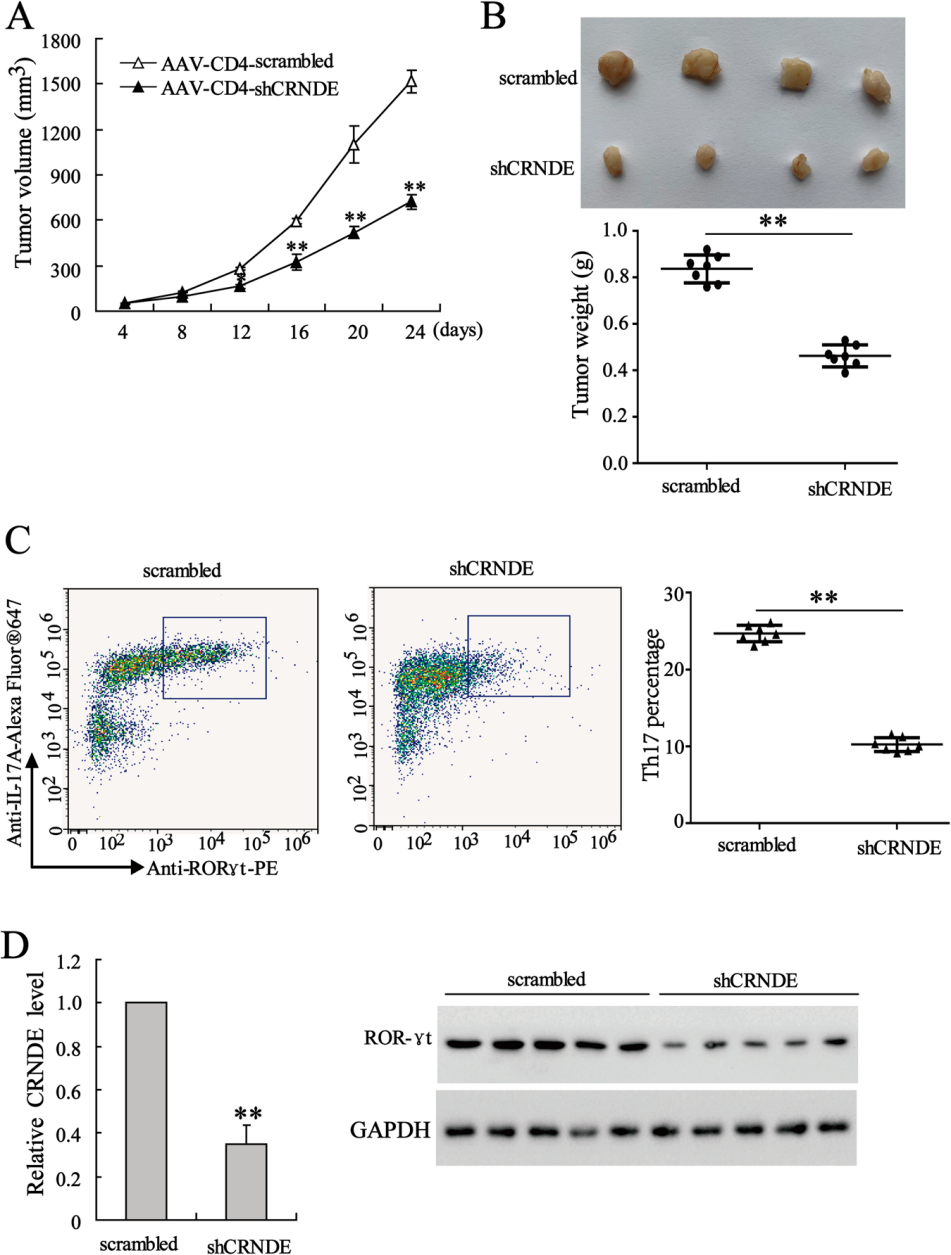
The silence of CRNDE-h in CD4^+^ T cells inhibited the CRC tumor growth in mice. The 8-week old male C57BL/6 mice were used to establish a tumor graft model by subcutaneous implantation of 0.2 mL CT-26 cells (5 × 10^6^ cells/mL). From the second week after implantation, mice received an intratumoral injection injected of AAV-CD4-shCRNDE-h or AAV-CD4-scrambled every week. Twenty-four days after injection, mice were sacrificed. **A** The tumor volumes were detected every four days. **B** The images and weights of dissected tumors. **C** The proportion of Th17 cells in the tumor-infiltrating T cells. **D** The expressions of CRNDE-h and RORγt in tumor-infiltrating T cells were detected by western blot and qRT-PCR. **p < 0.01 vs. AAV-CD4-scrambled.

To confirm the role of CRNDE in CRC tumor growth in vivo, we constructed AAV vectors (AAV-CD4-shCRNDE-h and AAV-CD4-scrambled), which were specifically expressed in CD4^+^ T cells. These AAV vectors were intratumorally injected into the tumor-transplanted mice at the second week after CRC cell (CT-26) inoculation. The tumor volumes were measured every four days. As shown in Fig. 7A, compared with AAV-CD4-scrambled, AAV-CD4-shCRNDE-h significantly reduced the tumor volumes in mice. Twenty-four days after injection, the mice were sacrificed, and their tumors were isolated. The representative images of tumors are presented in Fig. 7B, which shows that the injection with AAV-CD4-shCRNDE-h evidently decreased the tumors weights. Next, we isolated T cells that had infiltrated the tumors to detect the proportion of Th17 cells and the levels of CRNDE-h and RORγt. The flow cytometry assay showed that the AAV-CD4-shCRNDE-h injection reduced the proportion of Th17 cells in the tumor-infiltrating T cells (Fig. 7C). Meanwhile, qRT-PCR and western blot analysis revealed that the levels of CRNDE-h and RORγt were decreased in the AAV-CD4-shCRNDE-h injected mice (Fig. 7D). Collectively, the present results confirmed that knockdown of CRNDE-h in CD4^+^ T cells could inhibit CRC tumor growth in mice.

## Discussion

The increased Th17 cell number in tumor immune microenvironment is closely associated with the development and progression of CRC. Hence, it is necessary to explore the mechanism of Th17 cell differentiation in CRC. Herein, we innovatively demonstrated that CRC exosomes transmit CRNDE-h to CD4^+^ T cells to promote Th17 cell differentiation. Moreover, we have for the first time identified the mechanism by which CRNDE-h inhibits the ubiquitination and degradation of RORγt by inhibiting its binding to Itch, thereby promoting Th17 cell differentiation in CRC.

Tumor exosomes have become a hotspot in cancer research due to their important roles in tumor microenvironment. It has been reported that tumor exosomes can regulate Th17 cell differentiation in cancers. For instance, gastric cancer cell-secreted exosomes can promote Th17 cell differentiation under low glucose conditions^6^; also, CRC cell-derived exosomes can contribute to the differentiation of Th17 cells in the tumor transplantation model of CRC^16^. In the current study, we also found that the CRC exosomes promoted the differentiation of CD4^+^ T cells into Th17 cells. Although the role of CRC exosomes in Th17 cell differentiation has been determined in a previous study and in this work, the underlying mechanism remains largely unknown. Plenty of worthy evidence indicated that exosomes contain a plethora of RNA cargoes and that they carry out their roles by transmitting these RNA cargoes to adjacent cells^28^. Many studies have revealed that lncRNAs, such as lncRNA ARSR, lncRNA UCA1, and lncRNA TUC339, are the important RNA cargoes of tumor exosomes, and these lncRNAs mediate the role of exosomes in regulating drug resistance, tumor growth, and macrophage activation in many cancers^29–31^. We also found that the expressions of CRDNE-h in serum exosomes of CRC patients were significantly positively correlated with regional lymph node, metastasis and tumor size of the patients with clinicopathological characteristics (data not shown). This finding is consistent with previous reports, suggesting that exosomal CRNDE-h can not only serve as a marker for the diagnosis of CRC, but may also be related to tumor growth, invasion and metastasis^23–25^. On this basis, we conducted an in-depth study and found that the expression of CRNDE-h in serum exosome was positively correlated with the Th17 cell proportion in CRC patients, and this finding has led us to presume that CRC exosomes promoted Th17 cell differentiation by transferring CRNDE-h. Expectedly, we detected an abundance of CRNDE-h in CRC exosomes and found out that these CRC exosomes could transmit CRNDE-h to CD4^+^ T cells. The exosomes secreted by the CRNDE-h-depleted CRC cells could not promote Th17 cell differentiation compared with the normal CRC cell exosomes. By contrast, overexpression of CRNDE-h in CD4^+^ T cells increased the proportion of Th17 cells. These convincing lines of evidence demonstrated that tumor exosomes promote Th17 cell differentiation in CRC by transmitting CRNDE-h.

Numerous studies have demonstrated that CRNDE acts as a protooncogene in CRC by facilitating the proliferation, migration, metastasis, and chemoresistance^32,33^. Besides, a study has shown that many isoforms of CRNDE, including CRNDE-a, CRNDE-b, CRNDE-e, CRNDE-f, CRNDE-h, and CRNDE-j, were upregulated in CRC tissues and cells^22^. Among these isoforms, CRNDE-h is found to be stable in serum exosomes, and its increased level is linked to the poor prognosis of CRC^25^. In the present study, we verified that the CRC exosome-transmitted CRNDE-h promoted Th17 cell differentiation. Our in vivo experiment further suggested that the targeted silence of CRNDE-h in CD4^+^ T cells reduced the Th17 cell proportion in the tumor-infiltrating T cells and inhibited the tumor growth in the tumor-transplanted mice. These data further supported the oncogenic role of CRDNE in CRC. Moreover, a study has shown that the CRNDE-p isoform of CRNDE was abundant in the serum exosomes of CRC patients^34^. However, whether CRNDE-p or other CRNDE isoforms can mediate the role of CRC exosomes in regulating Th17 cell differentiation is largely unknown. More studies should be conducted to fill this knowledge gap.

RORγt, the key transcription factor for Th17 cell differentiation, can bind to the promoter of IL-17 to induce the secretion of IL-17 and the differentiation of naive CD4^+^ T cells into Th17 cells^26,35^. Considering this finding, we determined the effects of normal CRC exosomes or CRNDE-depleted CRC exosomes on RORγt expression and IL-17 promoter activity. Our results revealed that the CRNDE-depleted CRC exosomes could not increase RORγt expression and IL-17 promoter activity as normal CRC exosomes would, indicating that CRC exosomes increase RORγt expression and IL-17 promoter activity by transmitting CRNDE. Accumulating pieces of evidence have shown that CRNDE can regulate gene expression through various mechanisms. For example, Zheng et al. reported that CRNDE serves as a sponge of miR-384 to promote PIWIL4 expression in glioma^36^; Ding’s team reported that CRNDE binds to EZH2 and epigenetically suppresses the transcription of DUSP5 and CDNK1A by increasing the EZH2-mediated H3K27me3 levels on their promoters in CRC^37^; and Li et al. reported that CRNDE can increase the protein level of Smad3 by impeding the ubiquitination and degradation of Smad3 in abdominal aortic aneurysms^38^. The mechanism by which CRNDE-h, being an isoform of CRNDE, regulated the expression of RORγt greatly aroused our curiosity. To search for an explanation, we verified whether CRNDE-h could bind to RORγt. Our RIP and pull-down assays revealed that CRNDE could bind to the PPXY motif of RORγt. Considering that study has previously reported that the E3 ubiquitin ligase Itch could also bind to the PPXY motif of RORγt to induce the ubiquitination and degradation of RORγt in intestinal inflammation^27^, we studied whether CRNDE-h could regulate the Itch-mediated ubiquitination and degradation of RORγt. Our Co-IP analysis showed that CRNDE-h overexpression inhibited the binding of Itch and RORγt. The immunofluorescent staining results revealed that CRNDE-h overexpression attenuated the nuclear export of RORγt. Furthermore, RIP and RNA pull-down assays confirmed that CRNDE-h could not bind to Itch. More importantly, we found that the overexpression of CRNDE-h blocked the Itch-mediated ubiquitination and degradation of RORγt. Taken together, these results demonstrated that the CRC exosome-transmitted CRNDE-h promoted RORγt expression by hindering the Itch-mediated ubiquitination and degradation of RORγt.

In summary, this work suggested that the mechanism by which the tumor exosome-transmitted CRNDE-h contributed to Th17 cell differentiation involved inhibition of the Itch-mediated ubiquitination and degradation of RORγt in CRC. This finding not only expands our understanding of the mechanism of Th17 cell differentiation in CRC but also provides a new therapeutic target for CRC.

## Methods and materials

### Clinical samples

CRC patients and healthy controls were recruited from our center. All of the CRC patients were undergoing surgical therapy, and they all signed an informed consent. This study was approved by the Ethics Review Board of the First Affiliated Hospital of Zhengzhou University. Tumors, adjacent tumor tissues, and blood samples were collected from the CRC patients for analysis. The clinicopathological characteristics of the CRC patients are shown in Table 1.

**Table 1:** Clinicopathological characteristics in patients with CRC (*n* = 42).

Characteristics	Number of cases	Percent (%)
Age		
<60	22	52.4
≥60	20	47.6
Gender		
Male	25	59.5
Female	17	40.5
Tumor location		
Rectum	28	66.7
Colon	14	33.3
Tumor differentiation		
Well/Moderate	32	76.2
Poor	10	23.8
T stage		
T1–T2	11	26.2
T3–T4	31	73.8
Regional lymph node metastasis		
Negative	23	54.8
Positive	19	45.2
Distant metastasis		
No	33	78.6
Yes	9	21.4
Tumor size		
<5 cm	26	61.9
≥5 cm	16	38.1

### Isolation of tumor-infiltrating T cells

Tumor-infiltrating T cells were isolated from CRC tumors and from adjacent tumor tissues as previously described^6^. In brief, the tumors and adjacent tumor tissues were sheared into pieces, and then digested with 100 U/mL collagenase and 100 μg/mL Dnase. A 75 μm cell strainer was used to filter the isolated cells, and the Ficoll density gradient centrifugation method was used to separate the cells. The obtained mononuclear cells were resuspended in RPMI-1640 (Gibco, USA) containing 10% fetal bovine serum (FBS), and the T cells were positively sorted using Dynabeads^TM^ CD3 (Thermo Scientific, USA). In brief, the cells were incubated with dynabeads at 4 °C for 20 min. The supernatant was removed and the bead-bound CD3^+^ T cells were washed to be purified.

### Cell lines and culture

Human CRC cell lines (SW480, HT29, and LOVO), a human normal colon epithelial cell line (NCM460), a mouse CRC cell line (CT-26), and the 293 A cell line were purchased from ATCC (USA). The cells were cultured in Dulbecco’s modified Eagle’s medium (DMEM; Gibco) containing 10% FBS, and then incubated at 37 °C under 5% CO_2_. The human acute T-lymphocytic leukemia cell line (Jurkat) was purchased from ATCC (USA) and was cultured in RPMI-1640 supplemented with 10% FBS.

### Human T cell isolation and Th17 cell differentiation induction

Human peripheral blood mononuclear cells (PBMCs) were isolated from the buffy-coat of healthy donors through Ficoll density gradient centrifugation at room temperature for 30 min. The collected PBMCs were washed with PBS buffer. Naive CD4^+^ T cells were subsequently isolated from the PBMCs using a MagniSort Human CD4 Naive T Cell Enrichment Kit (Thermo Scientific) according to the manufacturer’s instructions. Naive CD4^+^ T cells were cultured in RPMI-1640 complete medium containing 100 IU IL-2 (R&D Systems, USA).

For Th17 cells differentiation, the healthy PBMCs were cultured in 24-well plates containing 25 μL/well Dynabeads™ Human T-Activator CD3/CD28 (Gibco), 100 ng/mL IL-6 (R&D Systems), and 20 ng/mL TGF-β1 (R&D Systems).

### Isolation and characterization of exosomes

The exosomes were isolated and characterized as previously described^39^. In brief, SW480, HT29, LOVO, and NCM460 cells were cultured in exosome-free DMEM complete medium for 48 h, and the culture supernatants were collected for exosome isolation. The exosomes in the cell culture supernatant or in the serum of CRC patients were isolated using Total Isolation Kits (Thermo Scientific) according to the manufacture’s specifications. Subsequently, the morphological characteristics of the exosomes isolated from the serum were observed under a transmission electron microscope (Thermo Scientific). The expression levels of the exosome-positive markers (CD63 and CD81) were detected using western blot analysis.

### Cell transfection

The small interfering RNA (siRNA) for CRNDE-h (si-CRNDE-h), the lentivirus (LV) vector carrying CRNDE-h (LV-CRNDE-h), and the LV carrying Itch were synthesized by GenePharma (China). A scrambled RNA was used as the negative control for si-CRNDE-h, and the LV carrying green fluorescent protein (LV-GFP) was utilized as the negative control for LV-CRNDE-h and LV-Itch. These vectors were transfected into cells using Lipofectamine 2000 (Invitrogen, USA).

### Flow cytometry

Tumor-infiltrating Th17 cells were detected by flow cytometry using BD PharmingenTM (San Jose, CA, USA) PE Mouse anti-Human RORγt, Alexa Fluor® 647 Mouse anti-Human IL-17A, and gated with FITC Mouse Anti-Human CD4. The number of IL-17A- and RORγt-positive cells on the gating of CD4^+^ cells was evaluated, and the frequency of Th17 cells was expressed as a percentage of the total CD4^+^ cells. Fluorescent-labeled isotype controls were used to determine background fluorescence and exclude unspecific binding.

### ELISA assay

The content of IL-17 in the culture supernatants of the PBMCs was detected using a Human IL-17 Quantikine ELISA Kit (R&D Systems) according to the manufacturer’s instructions.

### Real-time quantitative PCR (qRT-PCR)

The level of CRNDE-h was detected by qRT-PCR. Total RNA was isolated from the tumor-infiltrating T cells, CD4^+^ T cells, and induced PBMCs by using Trizol reagent (Invitrogen). After qualitative and quantitative analyses, 1 µg total RNA was utilized as a template to synthesize cDNA using High-Capacity cDNA Reverse Transcription Kits (Applied Biosystem, USA). The obtained cDNA was used to perform qRT-PCR with SYBR™ Green PCR Master Mix (Applied Biosystem). GAPDH was used as a housekeeping gene to normalize the expression of CRNDE-h. The expression levels of CRDNE in exosome secreted by NCM460 cells (NCM460-E), exosome secreted by SW480 cells (SW480-E), exosome secreted by HT29 cells (HT29-E), and exosome secreted by LOVO cells (LOVO-E) were calculated using 2^-CT^. Using 2^-ΔΔCT^ to calculate CRDNE level in tumor-infiltrating T cells.

### Western blot analysis

Total protein was extracted from the naive CD4^+^ T cells or from the tumor-infiltrating T cells by using RIPA lysis buffer (CWBIO, China) containing protease inhibitors. After being quantified by using a Pierce™ Microplate BCA Protein Assay Kit (Thermo Scientific), the protein was separated by SDS-PAGE and then transferred onto PVDF membranes. The PVDF membranes were then incubated with the primary antibodies; anti-RORγt (Abcam, 1/1000), anti-Itch (Abcam, 1/2000), and anti-GAPDH (Abcam, 1/2500) at 4 °C for 24 h; subsequently, the cells were incubated with a secondary antibody at room temperature for 1.5 h. The immune protein blots were visualized using a Pierce™ ECL Plus Western Blotting Substrate (Thermo Scientific).

### Immunoprecipitation assay

The RNA immunoprecipitation (RIP) assay was performed using a Magna RIP™ RNA-Binding Protein Immunoprecipitation Kit (Millipore, USA). In brief, the CRC-exosome treated human naive CD4^+^ T cells, 293 A cells, and tumor-infiltrating T cells were lysed with an RIP lysis buffer containing protease inhibitor and RNase inhibitor. A part of the cell lysate was used as input. The remaining cell lysate was divided into two parts and incubated with magnetic beads and with target antibodies (anti-Flag, anti-RORγt, anti-Itch, or anti-IgG) at 4 °C overnight. Then, the RNA samples were extracted from the immunoprecipitated products. The enrichment of CRNDE-h in the RNA samples was detected by qPCR. Anti-IgG was used as the negative control for the target antibodies.

For the co-immunoprecipitation (Co-IP) assay, 293 A cells were co-transfected with Flag-tagged RORγt (Flag-RORγt), Myc-tagged Itch (Myc-Itch), and LV-CRNDE-h. Then, the cells were lysed with Pierce IP Lysis Buffer (Thermo Scientific). The cell lysates were centrifuged at 4 °C, and 12,000 rpm for 10 min to obtain protein samples. Subsequently, protein samples were incubated with the antibodies of interest (anti-Flag and anti-Myc) at 4 °C for 2 h, and then incubated with protein A/G agarose (Thermo Scientific) at 4 °C for 24 h. The obtained complex was subjected to western blot analysis using anti-Myc or anti-Flag.

For the ubiquitination experiment, 293 A cells were co-transfected with Flag-RORγt, Myc-Itch, HA-tagged ubiquitin (HA-Ub), and LV-CRNDE-h, and then were treated with the proteasome inhibitor MG132. The cells were subsequently lysed with Pierce IP Lysis Buffer (Thermo Scientific), and then the cell lysates were centrifuged at 4 °C, 12,000 rpm for 10 min to obtain protein samples. The protein samples were incubated with anti-Flag and protein A/G agarose. The obtained complex was subjected to western blot analysis using anti-HA.

### RNA pull-down

First, the CRC-exosome treated human naive CD4^+^ T cells and the tumor-infiltrating T cells were lysed by a standard lysis buffer (Thermo Scientific). Then, CRNDE-h and its antisense RNA were labeled with biotin using a Thermo Scientific Pierce RNA 3′ End Desthiobiotinylation Kit (Thermo Scientific). Next, the biotin-labeled RNAs were separately incubated with streptavidin magnetic beads and cell lysates. The RNA-binding protein complexes were subsequently washed and eluted from the beads for further western blot analysis. The antisense RNA was utilized as a negative control for CRNDE-h.

### Luciferase reporter assay

The promoter sequence of IL-17 was inserted into the upstream luciferase-encoding gene of the pGL3-basic plasmid (Promega, USA) to establish a luciferase reporter gene. The luciferase reporter gene was then transfected into Jurkat cells using Lipofectamine 2000 (Invitrogen). After transfection, the Jurkat cells were treated with CRC-exosome. Finally, the luciferase activity was assessed by Dual-Luciferase Reporter Gene Assay Kits (Beyotime, China).

### RNA fluorescence in situ hybridization (FISH)

A cy3-labeled CRNDE-h probe was synthesized using a RiboTM lncRNA FISH Probe Mix (RiboBio, China). Human naive CD4^+^ T cells were treated with CRC cell-derived exosomes for the indicated times (4 and 24 h). Then, the location of CRNDE-h in the T cells was determined through RNA-FISH by using a Ribo^TM^ FISH Kit (RiboBio). In brief, the cells were fixed with 4% paraformaldehyde and then incubated with a transparent buffer. Subsequently, the cells were incubated overnight with 20 µL Cy3-labeled CRNDE-h probe (20 µM) at room temperature in the dark. The nucleus of the cells was stained with DAPI. Finally, the cells were observed under a confocal microscope (Leica, Germany).

### Immunofluorescent staining

Tumor-infiltrating T cells were transfected with LV-Itch or LV-GFP alone or co-transfected with LV-Itch and LV-CRNDE-h (or LV-GFP). The cells were seeded onto cover glasses and then incubated in a CO_2_ incubator. When the fusion rate reached 95–100%, the cells were washed with PBS three times, fixed with 4% paraformaldehyde for 20 min, permeabilized with 0.2% Triton X-100 for 10 min, and blocked with 5% BSA for 30 min. Subsequently, the cells were incubated with the antibody against RORγt at 4 °C overnight, and then incubated with a fluorescent-labeled secondary antibody at room temperature for 2 h in the dark. The nucleus of the cells was stained with DAPI. Finally, the cells were observed under a fluorescence microscope (Leica, Germany).

### Adeno-associated virus (AAV) recombinant vectors and tumor graft mouse model

The short hairpin small interfering RNA with the targeting sequence of 5′-GGTGTTAAGTGTGATGCTTCC-3′ for CRNDE-h (shCRNDE-h) was used. A scrambled sequence was used as a control for shCRNDE-h. The promoter of the human CD4 gene was cloned into AAV-CAG-GFP plasmids (Clontech, USA) to replace the CAG promoter. Then, the shCRNDE-h or the scrambled sequence was inserted into the upstream of the GFP reporter to generate recombinant AAV vectors (AAV-CD4-shCRNDE-h and AAV-CD4-scrambled).

Male C57BL/6 mice (8 weeks old) were used to establish a tumor graft model according to a previous publication, which was purchased from The Animal Experimental Center of Zhengzhou University^40^. All animal experiments were approved by the Ethics Committee of the First Affiliated Hospital of Zhengzhou University. For the tumor graft model establishment, all mice were subcutaneously implanted with 0.2 mL mouse CRC cells (CT-26, 5 × 10^6^/mL) in their right flanks. Starting on the second week after CT-26 cell implantation, seven mice received the intratumoral injection of 1 × 10^11^ AAV-CD4-shCRNDE-h particles every week, whereas the seven other mice received the intratumoral injection of 1 × 10^11^ AAV-CD4-scrambled particles every week. Tumors volumes were measured every four days. Twenty-four days after the viral particle injection, the mice were killed, and their tumors were excised and collected for further analysis.

### Statistical analysis

Statistical analysis was performed using Graphpad Prism 7.0. All data were expressed as mean ± standard deviation. Group differences were compared by Student *t-*test and one-way ANOVA whenever applicable. A *p*-value of < 0.05 represented statistical significance.

## Supplementary information

Supplemental Figure-1
