# Supplementary figures and images for: Tumor exosome promotes Th17 cell differentiation by transmitting the lncRNA CRNDE-h in colorectal cancer
Source: Cell Death Dis. 2021 Jan 25;12(1):123. doi: 10.1038/s41419-020-03376-y (PMC7835218; doi:10.1038/s41419-020-03376-y)

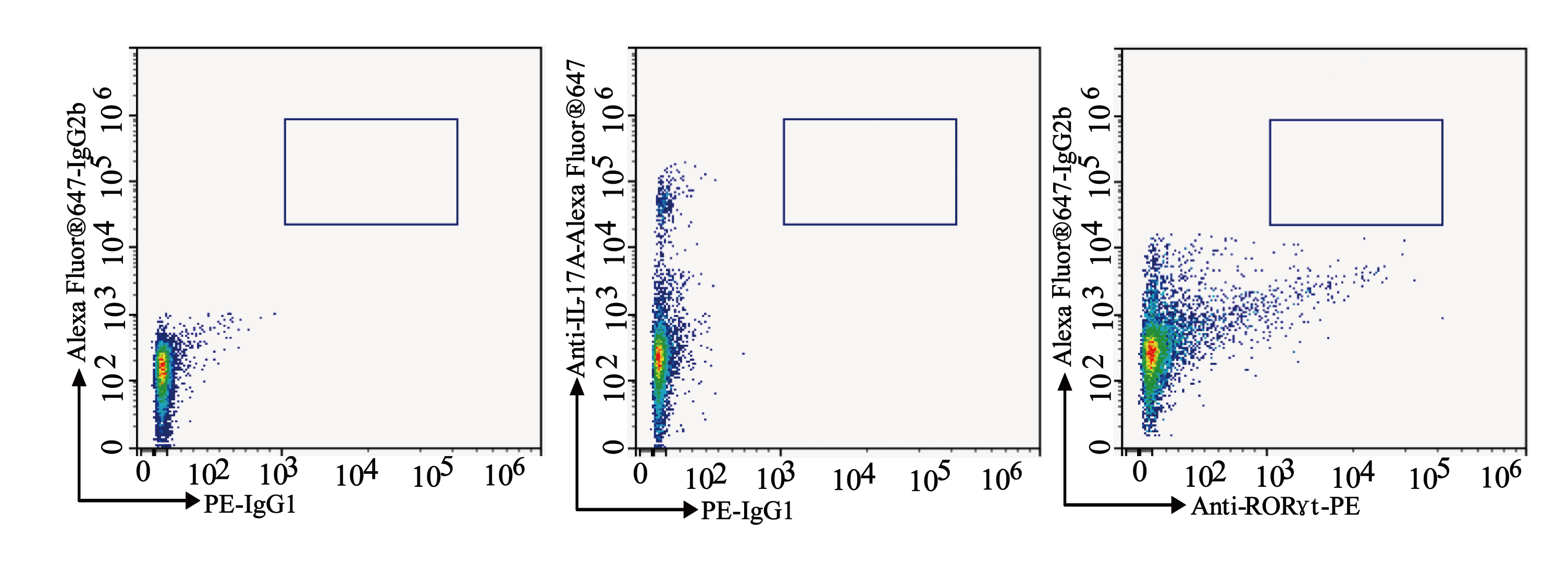

Supplement: Supplementary file 1 — Supplemental Figure-1 [file 41419_2020_3376_MOESM1_ESM.tif]
